# Supplementary figures and images for: Genome-wide identification and expression analyses of C2H2 zinc finger transcription factors in Pleurotus ostreatus
Source: PeerJ. 2022 Jan 5;10:e12654. doi: 10.7717/peerj.12654 (PMC8742544; doi:10.7717/peerj.12654)

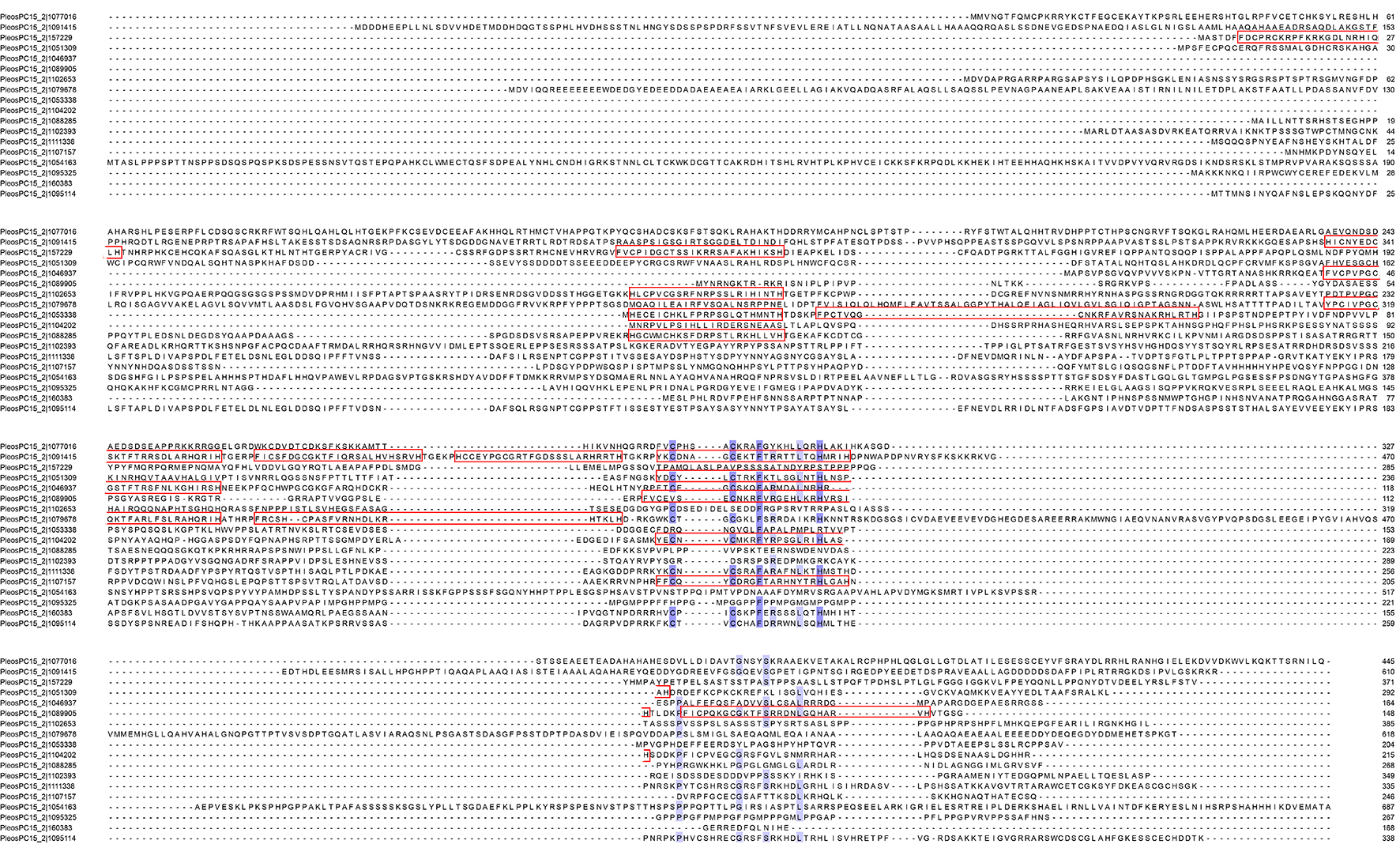

Supplement: Supplemental Information 1 — The C2H2 domains predicted on SMART (http://smart.embl-heidelberg.de) (with E-value < 1e−2) was enclosed by red box. [file peerj-10-12654-s001.png]
